# Supplementary material for: Disruptions in Effective Connectivity within and between Default Mode Network and Anterior Forebrain Mesocircuit in Prolonged Disorders of Consciousness
Source: Brain Sci. 2021 Jun 4;11(6):749. doi: 10.3390/brainsci11060749 (PMC8227204; doi:10.3390/brainsci11060749)
Supplement: Supplementary file 1 [file brainsci-11-00749-s001.zip › brainsci-1188919-supplementary.pdf]

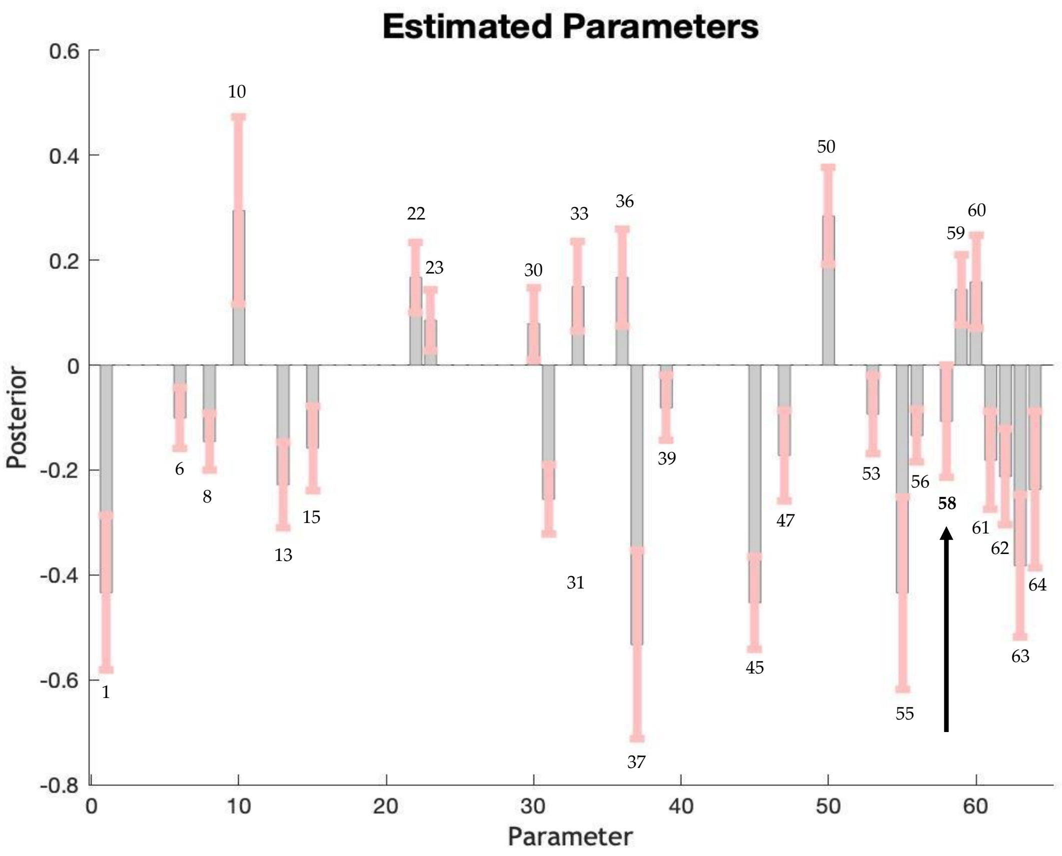

Parameter	From	To	Parameter	From	To
1	PCC	PCC	45	RTh	LTh
6	PCC	RTh	47	RTh	LStr
8	PCC	RStr	50	LStr	MPFC
10	MPFC	MPFC	53	LStr	LTh
13	MPFC	LTh	55	LStr	LStr
15	MPFC	LStr	56	LStr	RStr
22	LIPL	RTh	**58**	**RStr**	**MPFC**
23	LIPL	LStr	59	RStr	LIPL
30	RIPL	RTh	60	RStr	RIPL
31	RIPL	LStr	61	RStr	LTh
33	LTh	PCC	62	RStr	RTh
36	LTh	RIPL	63	RStr	LStr
37	LTh	LTh	64	RStr	RStr
39	LTh	LStr			

Figure S1. Posterior estimates after Bayesian model reduction (grey bars) and 95% Bayesian confidence intervals (pink lines). The parameters along the horizontal axis represent the connection number. The problematic parameter between the right striatum (RStr) and medial prefrontal cortex (MPFC) is indicated with an arrow (parameter 58).

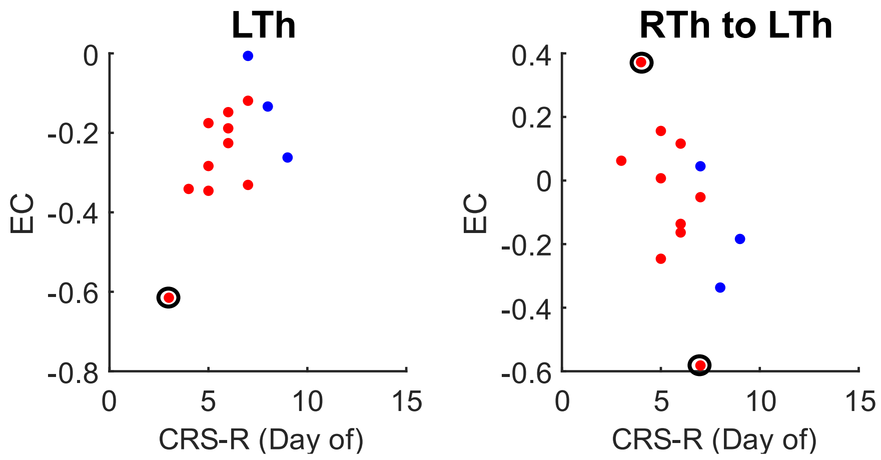

Figure S2. Correlations between effective connectivity (y-axis) in the Left thalamus (left) and the connection from right to left thalamus (right) and CRS-R (x-axis) on the day of the scan (N = 13). Note that these correlations are no longer significant when the outliers (circled red dot) are removed from the analysis. Red dots indicate vegetative state patients and blue show minimally conscious state. All connections (including self-connections) are displayed in Hz. EC, effective connectivity; CRS-R, coma recovery scale-revised; LTh, left thalamus; RTh, right thalamus.

Table S1. Correlation between CRS-R and effective connectivity in the regions and connections showing differences between PDOC and healthy controls.

		CRS-R on day of scan					Max CRS-R on week of scan				
		Kendall's Tau B	p-value	JZS-BF10	JZS-BF01	N	Kendall's Tau B	p-value	JZS-BF10	JZS-BF01	N
DMN	PCC to PCC	0.259	0.237	0.699	1.432	13	0.301	0.118	1.088	0.92	16
	PCC to RTh	-0.014	0.95	0.35	2.86	13	-0.177	0.358	0.486	2.058	16
	PCC to RStr	-0.423	0.053	2.199	0.455	13	-0.319	0.098	1.262	0.792	16
	MPFC	-0.123	0.575	0.408	2.452	13	-0.124	0.52	0.391	2.558	16
DMN to AFM		*-0.276*	*0.365*	*0.642*	*1.558*	*8*	*0.048*	*0.853*	*0.4*	*2.503*	*10*
	MPFC to LTh	0.095	0.663	0.384	2.607	13	0.071	0.713	0.34	2.945	16
		0.016	*0.944*	*0.363*	*2.756*	*12*	*0.02*	*0.92*	*0.328*	*3.045**	*15*
	MPFC to LStr	0.123	0.575	0.408	2.452	13	0.177	0.358	0.486	2.058	16
		0.048	*0.834*	*0.37*	*2.704*	*12*	*0.141*	*0.481*	*0.419*	*2.388*	*15*
	LIPL to RTh	0.041	0.852	0.355	2.816	13	-0.071	0.713	0.34	2.945	16
		0.048	*0.834*	*0.37*	*2.704*	*12*	*-0.153*	*0.447*	*0.439*	*2.28*	*15*
	LIPL to LStr	0.068	0.755	0.366	2.731	13	0.124	0.52	0.391	2.558	16
	RIPL to RTh	0.014	0.95	0.35	2.86	13	0	1	0.317	3.154*	16
	RIPL to LStr	-0.286	0.191	0.814	1.228	13	-0.106	0.582	0.37	2.704	16
	LTh to PCC	-0.204	0.35	0.538	1.859	13	-0.142	0.462	0.417	2.399	16

							-0.101	*0.614*	*0.371*	*2.696*	*15*
	LTh to RIPL	-0.204	0.35	0.538	1.859	13	-0.053	0.783	0.329	3.035*	16
		-0.463	*0.123*	*1.3*	*0.769*	*8*	*-0.327*	*0.176*	*0.909*	*1.1*	*11*
	**LTh**	**0.477**	**0.029***	**3.63**	**0.275**	**13**	0.266	0.168	0.828	1.207	16
		0.375	***0.105***	***1.331***	***0.751***	***12***	*0.183*	*0.362*	*0.497*	*2.012*	*15*
	LTh to LStr	-0.095	0.663	0.384	2.607	13	-0.018	0.927	0.318	3.14*	16
	**RTh to LTh**	**-0.45**	**0.04***	**2.804**	**0.357**	**13**	-0.337	0.081	1.477	0.677	16
		-0.35	***0.15***	***1.032***	***0.969***	***11***	*-0.258*	*0.218*	*0.72*	*1.388*	*14*
	RTh to LStr	-0.041	0.852	0.355	2.816	13	0.018	0.927	0.318	3.14*	16
		0.048	*0.834*	*0.37*	*2.704*	*12*	*0.04*	*0.84*	*0.333*	*2.999*	15
	LStr to MPFC	0.232	0.29	0.608	1.644	13	0.035	0.854	0.323	3.1*	16
							-0.02	*0.92*	*0.328*	_3.045*_	*15*
AFM to DMN	LStr to LTh	0.041	0.852	0.355	2.816	13	0.124	0.52	0.391	2.558	16
		0.145	*0.596*	*0.469*	*2.133*	*9*	*0.176*	*0.442*	*0.483*	*2.07*	*12*
	LStr	0.068	0.755	0.366	2.731	13	0.106	0.582	0.37	2.704	16
		-0.016	*0.944*	*0.363*	*2.756*	*12*	*0.06*	*0.762*	*0.342*	*2.924*	*15*
	LStr to RStr	-0.014	0.95	0.35	2.86	13	0.071	0.713	0.34	2.945	16
	RStr to MPFC	0.095	0.663	0.384	2.607	13	0.089	0.646	0.353	2.834	16
	RStr to LIPL	0.259	0.237	0.699	1.432	13	0.213	0.27	0.586	1.705	16
		0.272	*0.263*	*0.694*	*1.442*	*11*	*0.117*	*0.576*	*0.395*	*2.534*	*14*
	RStr to RIPL	0.204	0.35	0.538	1.859	13	0.177	0.358	0.486	2.058	16

	RStr to LTh	-0.041	0.852	0.355	2.816	13	-0.213	0.27	0.586	1.705	16
		-0.242	*0.355*	*0.598*	*1.671*	*10*	*-0.357*	*0.104*	*1.301*	*0.768*	*13*
	RStr to RTh	0.204	0.35	0.538	1.859	13	0.089	0.646	0.353	2.834	16
AFM							*0.04*	*0.84*	*0.333*	*2.999*	*15*
	RStr to LStr	0.314	0.152	0.964	1.037	13	0.355	0.066	1.743	0.574	16
		0.229	*0.342*	*0.58*	*1.726*	*11*	*0.38*	*0.067*	*1.742*	*0.574*	*14*
	RStr to RStr	0.259	0.237	0.699	1.432	13	0.124	0.52	0.391	2.558	16
		0.304	*0.184*	*0.855*	*1.169*	*12*	*0.081*	*0.687*	*0.354*	*2.822*	*15*

* $p < 0.05$, JSZ-BF10 > 3, JSZ-BF01 > 3; Italics represent results after removing outliers for each comparison. We highlight in bold the comparisons that indicated a significant correlation prior to removal of outliers, and in grey those that provided evidence for a lack of a correlation after removing outliers.

PCC, posterior cingulate cortex / precuneus; MPFC, medial prefrontal cortex; LIPL, left inferior parietal lobule; RIPL, right inferior parietal lobule; LTh, left thalamus; RTh, right thalamus; LStr, left striatum; RStr, right striatum.
